# Current insights in the preclinical study of palatal wound healing and oronasal fistula after cleft palate repair

**DOI:** 10.3389/fcell.2023.1271014

**Published:** 2023-10-12

**Authors:** Jiali Chen, Renjie Yang, Jiayi Yin, Bing Shi, Hanyao Huang

**Affiliations:** ^1^ State Key Laboratory of Oral Diseases and National Clinical Research Center for Oral Diseases, Department of Oral and Maxillofacial Surgery, West China Hospital of Stomatology, Sichuan University, Chengdu, Sichuan, China; ^2^ State Key Laboratory of Oral Diseases and National Clinical Research Center for Oral Diseases, Eastern Clinic, West China Hospital of Stomatology, Sichuan University, Chengdu, Sichuan, China

**Keywords:** mechanism, oronasal fistula, oral microflora, treatments, wound healing

## Abstract

Poor palatal wound healing after cleft palate repair could lead to unfavorable prognosis such as oronasal fistula (ONF), which might affect the patient’s velopharyngeal function as well as their quality of life. Thus, restoring poor palatal wound healing for avoiding the occurrence of ONF should be considered the key to postoperative care after cleft palate repair. This review provided current insights in the preclinical study of poor palatal wound healing after cleft palate repair. This review comprehensively introduced the animal model establishment for palatal wound healing and related ONF, including the models by mice, rats, piglets, and dogs, and then demonstrated the aspects for investigating poor palatal wound healing and related treatments, including possible signaling pathways that could be involved in the formation of poor palatal wound healing, the related microbiota changes because of the deformity of palatal structure, and the studies for potential therapeutic strategies for palatal wound healing and ONF. The purpose of this review was to show the state of the art in preclinical studies about palatal wound healing after cleft palate repair and to show the promising aspects for better management of palatal wound healing.

## 1 Introduction

Palatal wound healing happened after cleft palate repair and trauma and tumor excision (Majid, 2008; Sahoo et al., 2016; Alonso et al., 2019). Poor palatal wound healing could lead to oronasal fistula (ONF), resulting from extensive tension, infection, or hematoma (Hu and Rousso Joseph, 2020; Pollard et al., 2020). Oronasal fistula (ONF) was a common complication after cleft palate repair, which manifested as abnormal communication between oral and nasal cavities ranging from the alveolar process to the uvula (Honnebier et al., 2000; Mahajan et al., 2018; Alonso et al., 2019; Pollard et al., 2020), which could cause food reflux, unclear pronunciation and impaired velopharyngeal function, which affected the patient’s quality of life (Mahajan et al., 2018; Garg et al., 2019; Huang et al., 2023b). The incidence of ONF after cleft palate repair was up to 55% and the recurrence rate of ONF was up to 43% due to the cleft width, Veau types, and surgical techniques (Shankar et al., 2018; Sakran et al., 2022; Sakran et al., 2023a; Sakran et al., 2023b; Sakran et al., 2023c; Huang et al., 2023a). Thus, prevention of poor palatal wound healing should be an important mission to be completed.

In the situation of cleft palate repair, how to avoid poor palatal wound healing was always confusing, and the solutions were limited. Relaxing incision could help decrease the happening of poor palatal wound healing but might lead to further problems like inhibition of the maxillofacial growth (Becker and Hansson, 2013; Ogata et al., 2017; Mahajan et al., 2018; Liu et al., 2022). Thus, figuring out novel therapeutic strategies for preventing poor palatal wound healing and after cleft palate repair should be important in this field, and preclinical studies were essential. In this review, we first introduced the animal models that could be used for studying palatal wound healing, and then discussed current insights into the current aspects in the cause of poor palatal wound healing and related potential therapeutic strategies.

## 2 Animal models to study palatal wound healing

To understand palatal wound healing after cleft palate repair, animal studies were necessary. Here we showed the animal models that had been used in Table 1.

**TABLE 1:** Animal models for the creation of palatal wound.

Species	Strain	Method	Defect size	Site	Time for ONF formation	Study Ref
Mouse	Wild-type C57BL/6	1.5 mm cautery excise	1.5 mm	Hard palate	7 days	Ballestas et al. (2019)
	C57BL/6	1.5 mm biopsy punch	1.5 mm	Hard palate	10 days	Keswani et al. (2013)
	*Mus Musculus*/Swiss	1.5 mm biopsy punch	1.5 mm	Hard plate	5 days	Ferreira et al. (2021)
Rat	Wistar	4.5 mm biopsy punch	4.5 mm	Anterior palate	21 days	Suragimath et al. (2010)
	Sprague–Dawley	3 mm biopsy punch	3 mm	Hard palate	7 days	Zhu et al. (2015)
Piglet	Yorkshire	10 mm stainless steel biopsy punch	12 mm	Hard palate	2 weeks	Kirschner et al. (2006)
	“Schwaebisch Haellische”× “Pietrain”, hybrids	15 mm biopsy punch	>15 mm	Mid-hard palate	2 weeks	Rohleder et al. (2013)
Minipigs	Berlin	15 mm stainless steel biopsy punch	17 mm	Mid-hard palate	40 days	Kesting et al. (2010)
Dog	Beagles	6 mm biopsy punch	6 mm	Hard palate	2 weeks	Ayvazyan et al. (2011)

### 2.1 Mouse model

**FIGURE 1:**
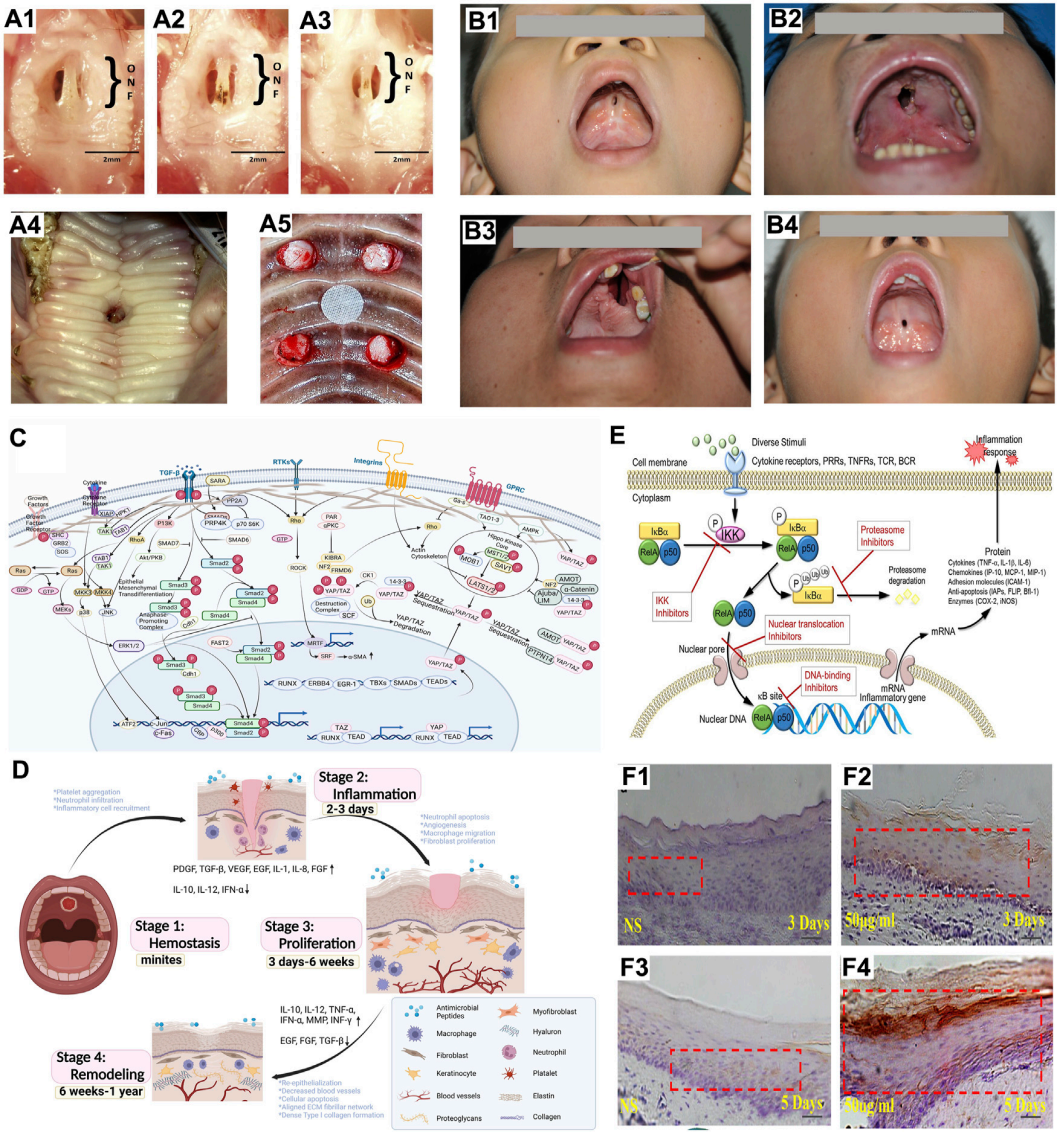
Studies on poor palatal wound healing and oronasal fistula after cleft palate repair **(A)** Animal models for studying poor palatal wound healing and oronasal fistula: **(A1–A3)** Changes in palatal wound in mice created with cautery over 7 days, **(A1)** Palatal wound on day 3, **(A2)** Palatal wound on day 5, **(A3)** Palatal wound on day 7 (Ballestas et al., 2019); **(A4)** Oronasal fistula on piglet’s palate 2 weeks after creation (Kesting et al., 2010); **(A5)** Four full-thickness defects on dog’s palate, mucosa and periosteum were removed and the palatal bone was exposed (Ayvazyan et al., 2011). **(B)** Common sites of ONF: **(B1)** A small ONF located at the anterior palate; **(B2)** A large ONF located at the anterior palate; **(B3)** ONF with alveolar cleft; **(B4)** ONF located at the junction of the hard and soft palate. **(C)** Mechanical force-related signaling pathways: Related signaling pathways activated by mechanotransduction were the Gridin/AKT signaling pathway, the TGF-β signaling pathway, the hippo signaling pathway and the RhoA/ROCK pathway (Created with BioRender.com). **(D)** The process of palatal wound healing: The process of palatal wound healing includes stages of hemostasis, inflammatory response, proliferation, and remodeling (Created with BioRender.com). **(E)** Four ways to reduce the inflammatory response by inhibiting NF-kB pathways, including inhibition of IKK kinase activity, inhibition of protease activity such as the 26S proteasome complex, inhibition of nuclear translocation prevented RelA and p50 from entering the nucleus, and inhibition of DNA binding to prevent the transcription process (Liu et al., 2017). **(F)** MMP-1 expression was higher in the group injected with *Lactobacillus reuteri* extracts (test group) on days 3 and 5 **(F2, F4)** than in the group injected with 0.9% NaCl (NS group) **(F1, F3)** (Han et al., 2019).

Take the approach of Ballestas et al. (2019) as an example (Figures 1A1–A3). An ophthalmologic cautery was used to create a 1.5-mm full-thickness mucosal injury on the hard palate. A similar approach was reported as well (Keswani et al., 2013). The differences were in the choice of mice and the tools used to create palatal wounds. A cautery excise was used to create a 1.5-mm full-thickness mucosal wound on the hard palate. In subsequent experiments, the formation of ONF was also confirmed by observing histological sections.

### 2.2 Rat model

A 3-mm biopsy punch was used to create a standardized circular wound on the central area of the hard palate, exposing the bare bone (Zhu et al., 2015). The wounds of the rats were recorded with stereoscopic microscope images 1 week after surgery.

### 2.3 Piglet model

A 10-mm stainless steel biopsy punch was used to create a full-thickness wound in the hard palate of piglets (Figure 1A4) (Kirschner et al., 2006). The edges of the bony palate and nasal septum were extended 2 mm to allow for mucosal reepithelialization about the periphery of the wounds. This method of creating wounds was also described in other publications, and the only difference is the size of the palatal wound (Kesting et al., 2010; Rohleder et al., 2013). ONF formation could be verified by morphological observation 2 weeks after the creation of the defect and was also verified when tissue sections were made for observation 5 weeks later.

### 2.4 Dog model

A biopsy punch was used to create standardized full-thickness wounds in the central area of the hard palate (Figure 1A5) (Ayvazyan et al., 2011). A rasp was used to remove the mucosa and periosteum to expose the bone. Photographs and tissue sections made during the experiment verified the final formation of ONF.

Overall, the differences between these methods were the sizes of the wounds and the instruments used. The two key points in creating a palatal wound capable of forming a final ONF were wound size and controlling asphyxia due to excessive bleeding during the procedure. A wound that was too small would not form a poor palatal healing. A wound that was too large would lead to difficulty in feeding, which would lead to excessive mortality and would not be conducive to subsequent experiments. The choice of instruments would determine the amount of intraoperative bleeding, which could affect death due to asphyxia. Currently, there were two main instruments, biopsy punch, and ophthalmologic cautery. The ophthalmic cautery was superior to the biopsy punch in reducing intraoperative bleeding because of its ability to stop bleeding at high temperatures, but it lacked in controlling the size of the wound and needed to be supplemented with other measuring tools.

## 3 Aspects of studies

### 3.1 Studies of the mechanism of palatal wound healing

As preventing poor palatal wound healing after cleft palate repair was the key to avoid ONF (Hu and Rousso Joseph, 2020; Wu et al., 2020; Li et al., 2021), the mechanisms for causing poor palatal wound healing should be explored. Extensive tension and infection have been found that contribute to the relatively high rate of poor wound healing (Ha et al., 2020; Hu and Rousso Joseph, 2020).

#### 3.1.1 Extensive tension

Clinically, a common site of poor palatal wound healing was the anterior palate, mainly due to the anatomical complexity of this area, particularly on the nasal side, where an inadequate issue often led to the failure of closure (Figures 1B1–B3). Another common site was the junction of the hard palate and soft palate, a site often considered to be the area of greatest tension (Figure 1B4) (Raoul and Ferri, 2007; Garg et al., 2019; Ha et al., 2020).Thus adequate tension reduction was the most crucial point in preventing poor palatal wound healing and tension-free suturing was a fundamental principle of cleft palate surgery (Agir et al., 2015; Elsherbiny and Grant, 2017).

Excessive tissue tension was an excessive mechanical force on the wound, which could affect wound healing (Huang et al., 2023a).The healing process of the oral mucosa included stages of hemostasis, inflammatory response, proliferation, and maturation (Guo and DiPietro, 2010). Excessive tissue tension might affect the proliferation and maturation stages. During the proliferation and maturation stages, the extracellular matrix (ECM) played a crucial role and was fundamental to the formation and function of soft connective tissues, which was affected by mechanical force (Leoni et al., 2015). Thus, we assumed that mechanotransduction could play an essential role in poor palatal wound healing after cleft palate repair.

The study about mechanotransduction in poor palatal wound healing was lacking, but we introduced some potential mechanisms that could be involved in poor palatal wound healing that need further investigation (Figure 1C). Transmembrane receptors on the cell membrane, mainly integrins, linked the extracellular to the intracellular, and intracellular integrin-linked proteins attached integrins to the cytoskeleton (CSK), and transmitted mechanical loads or signals within the cell (Hynes, 1992; Yin et al., 2022). The CSK stimulated by mechanical force went through complex morphological and biochemical changes and could trigger the transduction of mechanical signals into the cell (Hynes, 1992; Yin et al., 2022).

Related signaling pathways activated by CSK alterations were the Gridin/AKT signaling pathway, the TGF-β signaling pathway, the hippo signaling pathway, and the RhoA/ROCK pathway. It was found that Gridin family proteins were involved in regulating the activation of signaling pathways due to mechanical force (Enomoto et al., 2005), initiating the activation of the downstream signaling molecule Akt (ENOMOTO et al., 2006), and causing cytoskeletal reorganization (Bolcato-Bellemin et al., 2000a). Activated Transforming growth factor β1 (TGF-β1) induced the differentiation of epithelial cells into fibroblasts, inhibited collagenase release, and induced fibrillogenic effect (Bolcato-Bellemin et al., 2000b). Hippo signaling was an evolutionarily conserved pathway that controlled organ size by regulating cell proliferation, apoptosis, and stem cell self-renewal. YAP and TAZ were the two main downstream effector molecules of this pathway, which could induce the expression of genes that promoted cell proliferation and inhibited apoptosis (Toma et al., 2021).

#### 3.1.2 Infection and inflammation

Another common cause of poor palatal wound healing after the cleft palate repair was infection (Figure 1D). Sadhu et al. have reported that postoperative infection caused poor palatal wound healing, especially in adults (Sadhu, 2009). A methicillin-resistant *Staphylococcus aureus* infection with untreated candidiasis leading to poor palatal wound healing has also been reported (Palla et al., 2019). Unfortunately, the studies did not release the relationship between these infections and poor palatal wound healing in detail.

Infection could lead to inflammation, and inflammation could make wound healing more difficult and eventually lead to ONF. Thus, investigations on the mechanisms of infection and inflammation on poor palatal wound healing should be carried out and can be the potential therapeutic target for preventing poor palatal wound healing. Although the study about the roles and mechanisms of infection and inflammation in poor palatal wound healing was lacking, we introduced some potential mechanisms that could be involved in poor palatal wound healing.

In the inflammatory response, macrophages acted as the dominant cell type during the inflammatory phase of oral wound healing (Koh and DiPietro, 2011). M1 macrophages secreted cytokines, including interleukin-1 (IL-1), interleukin-6 (IL-6), fibroblast growth factor (FGF), platelet-derived growth factor (PDGF), epidermal growth factor (EGF) and TGF-β(Politis et al., 2016; Huang et al., 2022), which coordinated the movement of keratinocytes and fibroblasts towards the wound surface (Gallucci et al., 2000; Eming et al., 2007a). M2 macrophages promoted proliferation and healing and continued to secrete interleukin-10 (IL-10). M2 macrophages also helped to upregulate endogenous anti-inflammatory cytokine levels and downregulated pro-inflammatory cytokine levels previously secreted at the wound surface (Eming et al., 2007b). Thus, abnormal inflammatory response could result in the formation of chronic wound and lead to ONF.

### 3.2 Current studies on microflora of oronasal fistula

The oral mucosa was a barrier to microorganisms, and the oral cavity was a second habitat for microorganisms. Microorganisms played a crucial role in maintaining the dynamic balance, protecting the oral cavity, and preventing diseases (Wade, 2013). ONF was an abnormal structure in the oral cavity, which led to the abnormal airway structure and might alter the colonization of microflora in the oral cavity. We also presumed that specific microorganisms might colonize and secrete specific substances on the wound surface, leading to poor wound healing and the formation of ONF after cleft palate repair, making it more susceptible to associated diseases such as respiratory infections.

Related studies have focused on the relationship between cleft palate and dental caries (Cheng et al., 2007), periodontal disease (Costa et al., 2003; PERDIKOGIANNI et al., 2009), and changes in the oral microflora before and after cleft palate repair (Arief et al., 2005). Although no specific microbiological studies associated with palatal wound healing were found, the abnormal morphology and function of the oral cavity in patients with ONF created an oral environment that could be different from that of healthy individuals, just like cleft palate.

Therefore, these abnormal changes were likely to affect alterations in the number and composition of the oral microflora because of cleft and abnormal oral structure that could lead to the happening of postoperative poor palatal wound healing. These studies had a certain reference value. In a study on the oral microflora of infants with complete cleft lip and palate versus soft cleft palate, it was shown that infants with complete cleft lip and palate had a significant increase in the development of oral microflora with commensal and potentially pathogenic microorganisms (Machorowska-Pieniążek et al., 2017). Studies found that patients with cleft palate were more likely to develop periodontal disease, which was reflected in the higher plaque index, mild gingivitis, deeper periodontal pockets, and looseness of teeth (Costa et al., 2003; PERDIKOGIANNI et al., 2009). The correlation between cleft palate and caries was also reported, and it indicated that patients with cleft palate were more susceptible to caries (Cheng et al., 2007). However, another study demonstrated that the differences in the composition of the dental plaque microflora in patients with cleft palate had no statistical significance compared to healthy individuals (Funahashi et al., 2019). Changes in the oral microflora of cleft palate patients before and after surgery have also received attention. It was reported that the number of colonies was significantly lower in children with cleft palates after surgery (Arief et al., 2005).

Again, as described above in relation to the study of cleft palate and microflora, even though there was no study on poor palatal wound healing as well as ONF and related oral microflora, we could speculate that abnormal structures in the oral cavity of patients with ONF, such as oronasal connection, may predispose the patients to certain diseases, so it is important to figure out so that we can intervene further.

### 3.3 Therapeutic strategy for management of palatal wounds

#### 3.3.1 Study on growth factors and drugs

Nutrition of nerves, distribution of blood vessels and mechanical force could impact the normal palatal wound healing (Miron et al., 2017; Najeeb et al., 2017; Iglesias-Bartolome et al., 2018), so the drugs affecting these factors have been investigated.

During the wound healing process, platelets regulated the homeostasis phase through vascular obliteration and facilitated fibrin clot formation (Guo and DiPietro, 2010), and stimulated the proliferation and activation of cells involved in the healing process, such as fibroblasts, neutrophils, macrophages and stem cells. Growth factors such as platelet-derived growth factor (PDGF), adhesion molecules, and angiogenic factors were released and activated by platelets (Nurden, 2011). Therefore, platelet-rich fibrins (PRFs) regarded as a second-generation platelet concentrate was used to accelerate wound healing (Miron et al., 2016). It was demonstrated that PRFs could induce earlier complete wound epithelialization (Ozcan et al., 2017). In addition, PRFs contained an important component, fibrins, which acted as a bridging molecule that supplied a tridimensional matrix, so that fibroblasts and endothelial cells permeated within this fibrins network. Advanced platelet-rich fibrins (A-PRFs) also hasted palatal wound healing process in a prospective randomized study (Vanessa et al., 2020).

Recently, fingolimod hydrochloride (FTY720), an immunomodulatory drug was used to enhance the healing of palatal wound by increasing the accumulation of regenerating monocytes and macrophages on the wound surface (Awojoodu et al., 2013; Olingy et al., 2017). In animal studies of palatal wound healing, it was demonstrated that the use of FTY720 could promote wound healing and prevent poor palatal wound healing and the development of ONF after cleft palate repair (Ballestas et al., 2019). It also reported that FTY720 increased the expression of SOX2, which could increase the migration and proliferation of keratinocytes and leads to rapid wound healing (Ballestas et al., 2019). Studies have demonstrated that FTY720 reduced the expression of several pro-inflammatory interleukins, including IL-1, IL-4 and IL-6 and promoted the expression of the regenerative IL-10, which improved oral wound healing (King et al., 2013).

#### 3.3.2 Study on surgical techniques

For palatal wounds where ONFs have developed, surgeons often used a three-layer repair surgery, with the extra layer allowing for as much tension-free suturing as possible (El-Kassaby et al., 2014; Emodi et al., 2018). In recent years, many studies have used an acellular dermal matrix (ADM) to promoting palatal wound healing and repair ONF(Agir et al., 2015; Cassi and Massei, 2015). The use of ADM was first reported by Kirschner in 2006 (Kirschner et al., 2006). The ADM acted as a scaffold for host fibroblast migration and retained its basement membrane complex to facilitate attachment of the surface epithelium (Zhang et al., 2014). At the same time, it could solve the problems of insufficient tissue and scar formation (Agir et al., 2015). A study reported the use of collagen membrane to aid the closure of ONF and showed the effectiveness of good results of this surgery in repairing large ONF(Atherton and Boorman, 2016). However, it has been reported that it could not promote mucoperiosteum regeneration (Srivastava et al., 2001; Ophof et al., 2004; Liu et al., 2023).

#### 3.3.3 Study on other strategies

Electrical stimulation (ES) has been applied to treat many chronic wounds and some other diseases, showing promising results. In dentistry, it has been investigated in periodontitis (Kaynak et al., 2005; Tomofuji et al., 2013), orthodontic tooth movement (Qamruddin et al., 2017; Mistry et al., 2020) and bony defects (Kaynak et al., 2005). One study on the ES effect on the palate wound healing reported that it could accelerate the process of palate wound healing, especially in the early stages (Ferreira et al., 2021). The results showed that the level of IL-6, IL-10, TNF-α and VEGF was lower than the sham ES group (Ferreira et al., 2021). However, the mechanisms by which cells respond to ES were not figured out. One possible pathway to explain the reduced inflammation by ES could be the NF-kB suppression activity (Figure 1E) (Kaur et al., 2011; Liu et al., 2017). According to the NF-kB signaling, ES could contribute to increased angiogenesis, collagen matrix formation, contraction of the wound and reepithelization during the proliferative phase (Hunckler and De Mel, 2017).

The balance of oral pathogenic bacteria and probiotics also played a crucial role in maintaining the healthy status of the human host. It seemed that adjusting the balance of oral microflora was an effective way to promote wound healing. It was reported that the wound healing process was delayed due to the disturbances of microflora adhesion on the wound (Lindley et al., 2016). MSCs were considered a promising method that might promote tissue regeneration and wound healing due to the potential of multilineage differentiation and self-renewal properties (Park et al., 2018; Tsai et al., 2018). A study reported that the imbalanced oral microflora could impair the proliferation capability of mesenchymal stem cells (MSCs) and delay palatal wound healing (Figure 1F1–F4) (Han et al., 2019). *Lactobacillus reuteri* extracts were found to activate the potentials of GMSCs and enhance the wound healing process by regulating the PI3K/AKT/β-catenin/TGFβ1 pathway (Han et al., 2019). However, these studies only illustrated that unbalanced microflora could delay wound healing but did not show that balanced microflora would promote wound healing. A study revealed that the balance between oral pathogenic bacteria and probiotics could activate the functions of MSCs, which provided promising ideas and methods for the prevention and treatment of oral diseases (Han et al., 2020).

## 4 Summary

Poor wound healing after cleft palate repair would lead to ONF. In this review, we introduced the animal models for studying palatal wound healing and demonstrated the promising aspects for better managing palatal wound healing. Future research will focus on the molecular mechanisms of poor palatal wound healing, followed by the development of new materials and treatments.
